# Progress of Micro-Stimulation Techniques to Alter Pigeons’ Motor Behavior: A Review from the Perspectives of the Neural Basis and Neuro-Devices

**DOI:** 10.3390/brainsci14040339

**Published:** 2024-03-30

**Authors:** Mengmeng Li, Long Yang, Zhenlong Wang, Yuhuai Liu, Hong Wan, Zhigang Shang

**Affiliations:** 1 School of Electrical and Information Engineering, Zhengzhou University, Zhengzhou 450001, China; limengmeng@zzu.edu.cn (M.L.); longyang_zzu@163.com (L.Y.); ieyhliu@zzu.edu.cn (Y.L.); 2 Henan Key Laboratory of Brain Science and Brain Computer Interface Technology, Zhengzhou University, Zhengzhou 450001, China; wzl@zzu.edu.cn; 3 School of Life Sciences, Zhengzhou University, Zhengzhou 450001, China; 4 National Center for International Joint Research of Electronic Materials and Systems, International Joint-Laboratory of Electronic Materials and Systems of Henan Province, Zhengzhou University, Zhengzhou 450001, China

**Keywords:** altering pigeon motor behavior, pigeon robot, electrical stimulation, brain–computer interface

## Abstract

Pigeons have natural advantages in robotics research, including a wide range of activities, low energy consumption, good concealment performance, strong long-distance weight bearing and continuous flight ability, excellent navigation, and spatial cognitive ability, etc. They are typical model animals in the field of animal robot research and have important application value. A hot interdisciplinary research topic and the core content of pigeon robot research, altering pigeon motor behavior using brain stimulation involves multiple disciplines including animal ethology, neuroscience, electronic information technology and artificial intelligence technology, etc. In this paper, we review the progress of altering pigeon motor behavior using brain stimulation from the perspectives of the neural basis and neuro-devices. The recent literature on altering pigeon motor behavior using brain stimulation was investigated first. The neural basis, structure and function of a system to alter pigeon motor behavior using brain stimulation are briefly introduced below. Furthermore, a classified review was carried out based on the representative research achievements in this field in recent years. Our summary and discussion of the related research progress cover five aspects including the control targets, control parameters, control environment, control objectives, and control system. Future directions that need to be further studied are discussed, and the development trend in altering pigeon motor behavior using brain stimulation is projected.

## 1. Introduction

Animal motion can be effectively regulated through interventions in the nervous system, as shown by recent animal robot research regarding animal perception, movement, and decision-making mechanisms [1]. Driven by scientific and practical needs, animal robot research has experienced rapid growth, and has emerged as a thriving interdisciplinary field in recent years [2]. Flying animals have become pivotal model animals in this domain, owing to their innate advantages such as expansive activity range, low energy consumption, and adept concealment performance [3,4,5]. Pigeons, as a ubiquitous flying species, are known for their docile nature, ease of rearing, and widespread presence among human populations. They exhibit robust navigation and spatial cognitive abilities, alongside strong clustering and homing instincts. Moreover, they demonstrate exceptional long-distance load-bearing and sustained flight capabilities. These attributes position animal robots transported by pigeons as having promising applications in outdoor long-distance and flock-flight tasks [6,7].

So far, research on altering pigeon motor behavior using brain stimulation has predominantly focused on the principles of virtual punishment/reward (using stimulation of the specific brain region as ‘virtual’ punishments or rewards, delivered to freely roaming animals) [8,9] and midbrain motor region regulation. Brain–computer interface technology is used to apply specific stimuli to targeted brain regions (or nuclei) in pigeons, enabling control over their corresponding actions or completion of specific motion behaviors [8,10]. Benefiting from the significant advances in brain science, information science, control science, and materials science, along with the development of brain–computer interface technology, microelectronics technology, neural regulation technology, and microelectrode implantation technology, the structure and function of avian nervous systems has become clearer recent years [1,8]. The ability to analyze and simulate neural signals has gradually improved, and the exploration of remote communication transmission and long-distance stimulation technology has accelerated and deepened [3,9]. Furthermore, there has been a continuous improvement in the long-term effectiveness and biocompatibility of invasive implants [11,12]. These advancements have opened up possibilities for controlling pigeon motion from the perspective of precise functional localization of brain regions, reasonable encoding of stimulus patterns, and effective intervention in neural information. As a result, they have effectively promoted relevant research in the field of pigeon robotics [13].

The principle of pigeon robotics is applying micro-stimulation to relevant brain regions based on neurobiology in living pigeons to induce corresponding motion, turning them into manipulated creatures [1,8]. With their combination of animal body advantages and micro control systems, pigeon robots possess several unique characteristics. First, they exhibit autonomous intelligence, energy self-sufficiency, and high flexibility similar to living organisms. On the other hand, they also possess the high precision and controllability of micro systems [1]. With these advantages, pigeon robots can both mimic the excellent maneuverability of pigeons’ flying abilities and use the detection capabilities of their onboard sensors. By linking this research to practical applications, humans can use pigeon robots for information delivery, environmental monitoring, and reconnaissance rescue missions. This can enhance human society’s information monitoring and emergency response capabilities, providing strong support for the development and security of human society. As a result, pigeon robots hold immense scientific research and practical application value in various research fields such as bird brain structure and function, natural environment surveying and disaster warning, field reconnaissance, and search and rescue operations [14,15]. Therefore, the current review gives an overview of the scientific research and practical applications of pigeon robots, with focus on the relevant research findings in the control of pigeon motion, while highlighting the recent advances and future prospects.

## 2. Altering Pigeon Motor Behavior Using Brain Stimulation

**Figure 1:**
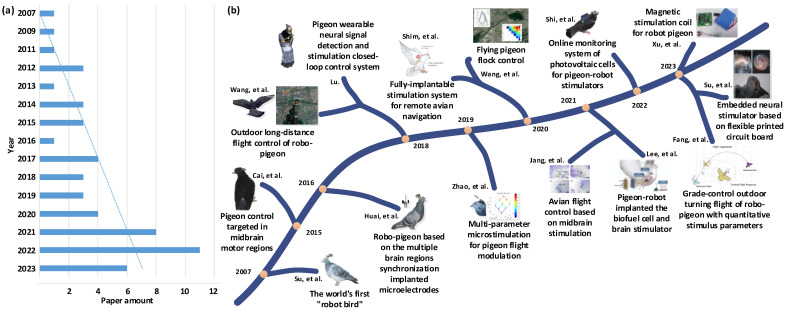
Research advances of altering pigeon motor behavior since 2007. (**a**) Number of published research articles; (**b**) the representative research work [5,6,7,10,16,17,18,19,20,21,22,23,24,25].

Altering pigeon motor behavior using brain stimulation is a multidisciplinary research field. Since 2007, when the Prof. Su Xuecheng’s team from Shandong University of Science and Technology (SDUST, Qingdao, China) successfully released the world’s first ‘robot bird’ using the pigeon as the model animal, altering pigeon motor behavior using brain stimulation has gained continuous attention from many research institutions all over the world. Subsequently, a series of remarkable research results has been published. By searching literature databases such as Web of Science, Google Scholar, and China National Knowledge Infrastructure (as of December 2023) and conducting secondary manual verification using keywords such as “pigeon robot”, “pigeon motor control using brain stimulation” and “altering pigeon motor behavior using brain stimulation”. The taxonomy used in our review includes key search terms, languages, and paper sources. Initially, we obtained totally 219 results from all sources. Our exclusion criteria were only mentioning the above terms or not involving them in the core of the paper. On these criteria, 166 of the papers were excluded from this review. Of the remaining 53 results (27 English papers and 26 Chinese papers), 31 were from journals, 14 from theses, and 8 from conference proceedings. The publication status of related research was obtained (Figure 1a). It can be seen that although the current number of relevant papers is not large, the overall number of papers shows a significant increasing trend year by year. Especially after 2017, several teams from SDUST, Nanjing University of Aeronautics and Astronautics (NUAA, Nanjing, China), Zhengzhou University (ZZU, Zhengzhou, China), Seoul National University (SNU, Seoul, Republic of Korea), and HuangHuai University (HHU, Zhumadian, China) have carried out a series of related research projects and reported their research findings (Figure 1b), achieving gratifying results [6,16,17].

Overall, the technology altering pigeon motor behavior using brain stimulation has made continuous breakthroughs in various aspects including diversification of control targets, refinement of control parameters, increasing complexity of control environments, flock-oriented control, multi-functionality of control systems, and so on. This has led to sustained improvements in control effectiveness towards more accuracy, longer distances, and more stable outcomes. These advancements have also laid a stronger foundation for future animal control and animal robot research.

### 2.1. The Neural Basis for Altering Pigeon Motor Behavior Using Brain Stimulation

Research on the neuro-motor control functions is an important cornerstone for achieving behavior control. The study of avian brain function can be traced back to the early 19th century. In 1809, Rolando first used cerebellar ablation to study the function of the avian cerebellum and found that damage to the cerebellum significantly affected the posture and motor-related functions of birds. Ferrier’s avian cerebellar electrical stimulation experiments in 1886 demonstrated that electrical stimulation could induce behaviors such as head turning, wing flapping, or leg bending in birds on the corresponding side [26]. In the 20th century, with the continuous deepening of relevant research, researchers gained a clearer understanding of the distribution and composition of avian brain regions related to altering pigeon motor behavior using brain stimulation. In 1958, Raymond [27] from the University of Michigan used implanted electrodes and constant current electrical stimulation to study the brain function of chickens and pigeons in a wakeful state during perching, walking, standing, and flying. In the 1980s, Steeves and his team [28,29,30] from the University of British Columbia conducted a significant amount of work in the field of avian brain function research using various methods including lesion, electrical stimulation, and chemical stimulation.

**Figure 2:**
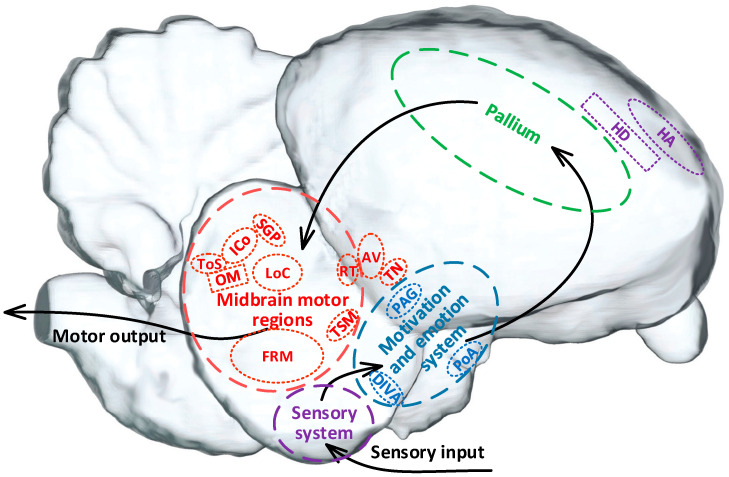
The motion-related neuronal system of a pigeon. Note. HA, hyperstriatum accessorium. HD, hyperstriatum dorsal. DIVA, dorsalis intermedius ventralis anterior. PoA, posterior pallial amygdala. PAG, periaqueductal gray. ToS, torus semicircularis. ICo, nucleus intercollicularis. SGP, substantia griseaet fibrosa periventricularis. FRM, formatio reticularismedialis mesencephali. LoC, locus ceruleus. RT, nucleus rotundus. OM, tractus occipito-mesencephalicus. TN, nucleus taeniae. TSM, tractus septo-mesencephalicus. AV, archistriatum ventral.

Thus, it has been shown that pigeons rely on multi-level hierarchical neural systems in the brain to complete the process from sensory input to motor output. This process involves the sensory system (purple marking), motivation and emotion system (blue marking), pallium (green marking), and midbrain motor regions (red marking) (Figure 2). These brain regions work together to support the execution of motion behaviors [1,8]. Among them, the sensory system is primarily responsible for receiving and integrating internal and external sensory information, which is then transmitted to the motivation and emotion system for fusion and motion motivation planning [31]. The pallium serves as a higher-order cognitive and decision-making center related to motion [32], responsible for formulating motor strategies [33,34]. The midbrain motor regions are responsible for the bidirectional projection of motion information, transmitting coordinated motion commands to various relevant functional nuclei to initiate, maintain, and stop motion behaviors [1,10,35].

According to the differences in the stimulation target, the control strategies for pigeon motion can be roughly divided into three categories (Table 1). One is the control strategy combined with behavioral training and with the specific regions in the sensory system as the target including the hyperstriatum accessorium (HA) and the hyperstriatum dorsal (HD) [36]. However, early electrical stimulation-based experiments targeting these regions did not achieve the expected effect in altering pigeon motor behavior, mainly due to the interference of the integration of various sensory input signals [8].

The second is the category targeted in the specific regions in the motivation and emotion system or pallium that are related to motion selection and decision-making to induce motives or virtual emotions for control. The typical regions include the dorsalis intermedius ventralis anterior (DIVA), the posterioramygdala (PoA), and the periaqueductal gray (PAG) [5,8,37,38]. Stimulation in these regions has successfully induced expected pigeon motion such as takeoff, hovering, left turn, right turn, and forward motion, bringing about a new era in the study of altering pigeon motor behavior using brain stimulation. However, the precision, stability, and reliability of the control still need to be further improved due to the complexity of the involved nuclei and circuits.

The third is the category targeted in the midbrain motor regions responsible for coordinating the movement process to directly control motion. These numerous related nuclei include torus semicircularis (ToS) [39], nucleus intercollicularis (ICo) [10,39], substantia grisea et fibrosa periventricularis (SGP) [39,40,41], formatio reticularismedialis mesencephali (FRM) [11], locus ceruleus (LoC) [10], as well as the nucleus rotundus (RT), tractus occipito-mesencephalicus (OM), nucleus taeniae (TN), tractus septo-mesencephalicus (TSM), and archistriatum ventral (AV) [18]. The stimulation-based control targeted in the midbrain motor regions bypasses the complex upward processes of information integration, judgment, and motion planning decision-making, significantly reducing potential interference during the control [10]. However, further research is needed to explore the deep mechanisms of behavior control, design appropriate stimulation patterns, and set precise stimulation parameters due to the dense distribution and difficult localization of relevant nuclei within the midbrain, as well as the fact that stimulation of multiple regions here often induces similar behavior.

To understand the needs of future research on altering pigeon motor behavior using brain stimulation, some details are given to clarify the neural mechanisms of the multiple neural subsystems mentioned above. Firstly, deeper investigation of the connections between these subsystems, especially the various nuclei closely associated with motion, is required. This is also the foundation for precise brain region localization to induce specific behavior. Secondly, a more detailed interpretation is needed on how these subsystems encode motion-related neural information. This is of significant importance for decoding motion-related neural information, simulating spontaneous neural activity in pigeons, and designing stimulation signals. Lastly, the transmission relationships (including transmission order and interactions) of motion-related neural information within each subsystem need to be further elucidated. This will help to develop more reasonable and coordinated sequential stimulation strategies considering the hierarchical and multi-level control processes of motion comprehensively.

### 2.2. The Composition and Functions of the System for Altering Pigeon Motor Behavior Using Brain Stimulation

**Figure 3:**
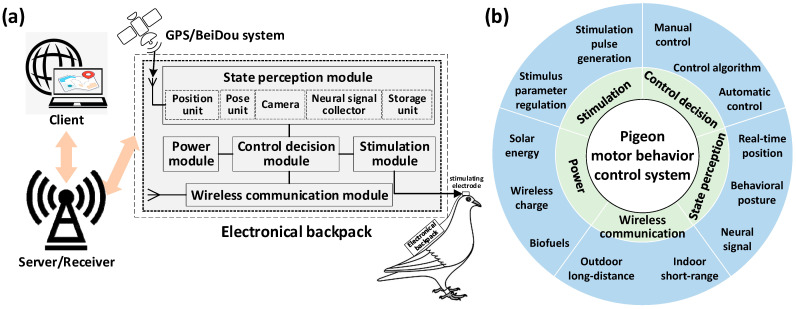
The system altering pigeon motor behavior using brain stimulation. (**a**) System configuration; (**b**) main functions integrated by the system.

The current research on altering pigeon motor behavior using brain stimulation mainly involves the use of artificial devices to generate microcurrents, stimulating specific nuclei in the pigeon’s brain to induce behavioral changes (including takeoff, left/right turn, avoidance and so on) and successfully achieve the control purpose [1,8]. The control system used to generate microcurrent stimulation generally consists of four components: microelectrodes implanted in specific regions of the pigeon’s brain, an electrical backpack carried by the pigeon, a server or receiver responsible for data communication, and a client for stimulation control and motor trajectory display (Figure 3a). The client provides an interface for operators to issue stimulating command. The electrical backpack generates specific stimulation pulses after receiving the commands, which act on the pigeon’s brain through the microelectrodes to control its motion. At the same time, the electrical backpack collects real-time information and provides feedback to the client for the operator to monitor the pigeon’s motion state. To achieve the above control task, the system used generally needs to integrate major functions including state perception, stimulation generation, control and decision-making, wireless communication, and power supply (Figure 3b).

#### 2.2.1. The State Perception Module

The role of the state perception module is to obtain real-time status of pigeons, to execute control of the current position, posture, or neural dynamic information. For indoor control tasks, existing research mainly relies on real-time position information recorded by motion tracking systems [18] or visual observations of pigeon behavior by operators [12]. In outdoor open environments, especially in long-distance control tasks, most studies choose to use a global positioning system (GPS) to obtain real-time position status of pigeons [6,42] to overcome the limitation of the infeasible visual observation and the difficult construction of motion capture systems. In addition, posture [43,44] and neuroelectrophysiological recordings [44,45] can provide more rich information than positional recordings. Therefore, how to integrate multi-modal state information to achieve automatic navigational control of pigeons still requires further in-depth study in the future research aimed at outdoor environments.

#### 2.2.2. The Stimulation Module

The stimulation module is used to apply electrical stimulation signals to specific target regions in the pigeon’s brain. Through simulating the spontaneous neural signals in the specific brain regions, we can encode the stimulation signals and apply them into these regions to achieve behavior control objectives. This kind of neural electrical stimulation essentially works by inducing similar neuron responses in the specific brain regions. Therefore, it is necessary to consider the discharge characteristics of neurons within specific brain regions to design appropriate electrical stimulation signal coding patterns to ensure effective long-term response [1]. Previous studies on electrical pulse stimulation systems applied to freely moving small animals found that the influencing stimulation parameters of the control effect included pulse amplitude, duration, frequency, and number [46]. Subsequent studies further explored the behavioral control effects corresponding to different settings of the stimulation parameters [16,38], in which multi-parameter continuously adjustable stimulation generators provided the necessary stimulation input. In the future, electrical stimulation design for altering pigeon motor behavior using brain stimulation should fully consider appropriate combinations of multiple parameters to achieve better control effects.

#### 2.2.3. The Control Decision Module

The control decision module relies on control algorithms to implement behavioral control of animals. According to whether human participation is required, the pigeon behavioral control system can be divided into two categories: manual control systems and automatic control systems. Manual control systems are mainly used in indoor scenes during which the operator determines when to apply control commands by observing the pigeon behavior [47]. This type of system is relatively flexible but usually has high uncertainty. Automatic control systems have great potential for long-distance outdoor control tasks in which the control commands are determined by computer programs. This type of system generally relies on specific perception resolution and control algorithms for an animal’s behavioral state [42,48], with less restriction in experimental scenarios. However, due to difficulties in real-time acquisition of pigeon motion states in free-flight scenarios and limitations in the pigeon’s own capacity to carry heavy equipment, breakthroughs are still needed to enrich current long-distance outdoor automatic control research on pigeons.

#### 2.2.4. The Wireless Communication Module

A wireless communication module is mainly used for real-time feedback of pigeons’ state information and transmission of stimulation commands [49]. In indoor experimental scenarios with closer communication distances, communication methods for control mainly include infrared [50], radio frequency transceiver chips [51], Bluetooth [52], Zigbee mesh networks [53], etc. The working distances of these above methods generally do not exceed 500 m, which severely limits the mobility of pigeon robots. Communication methods based on outdoor civilian communication network base stations can effectively overcome the limitations of distance and expand the working space of the control system [54], but they also face issues of high power consumption and transmission latency. In environments with poor network communication conditions, distributed and collaborative manners for data collection, processing, and network communication can help achieve autonomous behavioral control based on the biological sensor network constructed using pigeon flocks [55]. In future research, more efforts should be made to address the challenges of remote long-term data communication and stable transmission.

#### 2.2.5. The Power Module

The power module providing stable energy is the heart of the control system. The system altering pigeon motor behavior using brain stimulation usually adopts a lithium battery power supply. The load capacity of pigeons and the energy density of lithium batteries are the main factors limiting the working time of the system. In the current stage, where it is impossible to increase the energy density of power sources, convenient and long-lasting charging methods are necessary to ensure the system’s long-term operation. In recent years, various systems including implantable wireless charging stimulators [17], biofuel cell charging stimulators [19], stimulators charged by high-performance single-crystal silicon photovoltaic cells [20], and ultra-thin organic solar modules [56] have been gradually developed and used in animal robot control. However, the output power of biofuel and solar charging devices is relatively low at present. The challenge resulting from this is that they cannot meet practical needs and only enable basic control of animal robots. Therefore, the development of efficient power modules suitable for controlling pigeon motion still needs breakthroughs.

## 3. Recent Advances and Future Prospects in Altering Pigeon Motor Behavior Using Brain Stimulation

**Figure 4:**
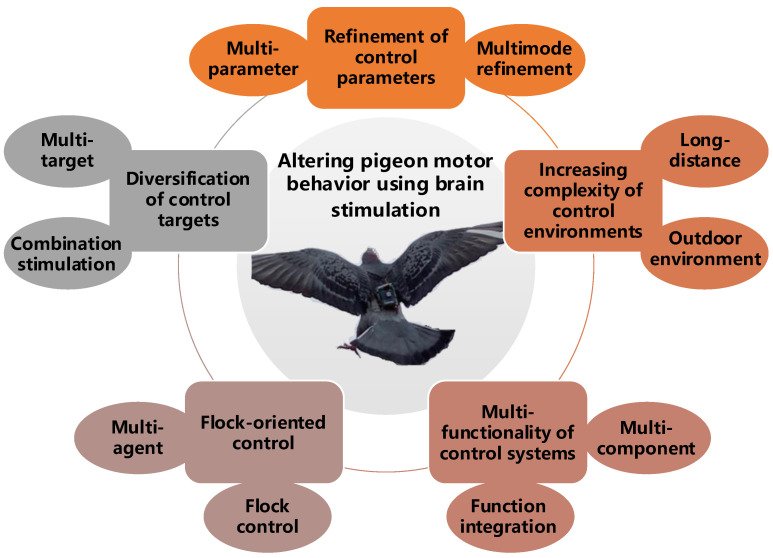
Research topics on altering pigeon motor behavior using brain stimulation.

Thanks to significant advances in the related neuroscience research of pigeon motion and the gradual maturation of control systems, there have been noteworthy achievements in this field. In this section, we combine the research progress and future prospects of altering pigeon motor behavior using brain stimulation to classify and review the latest research findings in recent years (Table 2). Five aspects are the focus in our review: control targets, parameters, environments, subjects, and systems (Figure 4).

### 3.1. Diversification of Control Targets

The earliest attempt to induce pigeon motion using electrical stimulation targeted the cerebellum, as birds have a well-developed cerebellum that plays an important role in maintaining motion coordination and body balance [26]. Later research further expanded the scope to more widely distributed motor-related brain regions (Table 2, column ‘Targets’). The team led by Su from SDUST, who first conducted a series of pigeon robot studies in China, initially chose brain regions related to fear and pain perception as the targets to induce specific motion in pigeons [5]. Then, the teams led by Dai from NUAA [10] and Kim from SNU [18] conducted further research targeting more regions in the midbrain motion area, clarifying the relationship between the nuclei in pigeons’ midbrain and their motor functions. Their efforts provided more solid experimental evidence for the diversification of control targets to control pigeon motion.

However, the existing research on altering pigeon motor behavior using brain stimulation still largely relies on single-site control, which has certain limitations. In 2016, Su’s team [21] proposed a method of synchronously implanting multiple microelectrodes in different brain regions based on their previous research. They implanted microelectrodes simultaneously by calculating the distance and depth differences between multiple regions to reduce the potential errors caused by single-electrode implantation, laying the foundation for multi-target stimulation-based control. In 2021, Wang’s team from ZZU [57] developed a customized device for multi-site microelectrode implantation in pigeons according to the positional relationships of specific nuclei using Altium Designer 16.0.6 software. This multi-site bio-machine interface, equipped with lightweight interfaces and communication modules and combined with electrical stimulation at different sites, can reduce the difficulty of implantation surgery and elicit a wider range of behavioral combinations in pigeons, providing the necessary application basis and technical support for future research on pigeon flight control.

Although the mentioned studies have made meaningful attempts in the field of multi-target stimulation-based pigeon motor behavior control using brain stimulation, the relationship and differences between behaviors induced by different targets within specific neural systems (details shown in Table 1) still require further exploration. In addition, there are still significant challenges to overcome in designing more precise and long-lasting combined stimulation patterns to achieve more continuous and stable behavior control effects on the basis of a deeper understanding of the neural circuit mechanisms of altering pigeon motor behavior using brain stimulation. In future research, it is crucial to further clarify the relationship between neural targets related to pigeon motion, avoid the uncertainty caused by the diffusion of micro-stimulation signals between multiple targets (especially close-range targets), and explore effective combined stimulation patterns for precise control.

### 3.2. Refinement of Control Parameters

Different types of stimulation signals applied to specific brain regions can be used to induce the expected motion of pigeons. Due to the similarity between artificial electrical stimulation signals and spontaneous neural activity, along with the mature easy-to-use devices and control platforms, electrical stimulation-based control with strong sensitivity and high repeatability remains the mainstream mode in current research [58]. In early studies, single constant voltage signals were used in the design of electrical stimulation parameters. However, the impedance of the stimulating electrodes increases over time with implantation [59], resulting in the decrease of the current and reduction of the stimulation effect. Therefore, Su’s team [8] proposed that constant current signals should preferably be selected as the simulated stimulation signals to maintain consistency with the current characteristics of intrinsic neural information in 2012. Subsequent research found that externally introduced electrical stimulation cannot fundamentally maintain consistency with the inherent current characteristics of the nervous system. Single stimulation parameters including pulse amplitude, frequency, or width may not induce the same motion. Using this kind of single-modal stimulation, it is also difficult to achieve precise and sensitive control of pigeon motion and it cannot adapt to individual differences [1]. Therefore, subsequent research has enriched the design of multimodal stimulation, and serialized and timely stimulation combinations have gradually become a universal pattern for fine-grained control of pigeon motion (Table 2, column ‘Parameters’).

In 2015, Su’s team [38] developed a multimodal electrical stimulation system that can emit multi-mode, non-steady-state TTL (transistor–transistor logic) biphasic pulses, achieving reliable control of typical pigeon motion. The control effect indicates that multimodal stimulations exhibit better neural adaptability than single-modal ones. In 2018, Wang et al. [6] designed a hierarchical stimulation method based on single stimulation, periodic stimulation, and multi-period stimulation to ensure the effectiveness of altering pigeon motor behavior, achieving good results in outdoor flight control of pigeons. In 2019, Wan and Shang’s team from ZZU [16] quantitatively analyzed the relationship between the amplitude, frequency, duty cycle of the electrical stimulation pulse, and the flight behavior of pigeons. They found a significant nonlinear correlation between these parameters and flight behavior, providing necessary reference for designing precise control of pigeon motion based on multi-modal stimulation combinations. In the same year, Yang [60] designed a multi-modal neural stimulation method to cope with the decrease in controlled sensitivity caused by the fatigue generated by constant electrical stimulation in the nervous system. The results showed that random-changing multi-modal stimulations had shorter time consumption and more stable control effects for robotic pigeons compared with single-modal ones. In 2023, Dai’s team [22] quantitatively studied the influence of electrical stimulation frequency, duration, and interval on robotic pigeon control in outdoor turning flight. They found that the turning flight of pigeons can be controlled in a graded way by selecting different stimulation variables, which is helpful for further optimizing the stimulation strategy of robotic pigeons to achieve precise control of their outdoor flight behavior.

The abovementioned studies have made exploratory preliminary research on the relevant stimulation parameters for controlling pigeon behavior. However, the control effects are still influenced by the combination of parameters, numerical settings, and individual tolerances when considering the demands for precise and reliable applications. Therefore, there is still a need for in-depth research on customized parameterization of pigeon behavior control tailored to individual needs. In addition, using stimulation signals of other modalities, such as light [61,62] and magnetic stimulation [23,63] to control avian behavior, and further exploring the control effects corresponding to different parameters, as well as designing multi-modal parameter design schemes for fine-tuned control requirements, will also be valuable research directions.

### 3.3. Increasing Complexity of Control Environments

Early research on pigeon flight control was mostly conducted in indoor environments (Table 2, column ‘Environments’), mainly due to limitations in the communication transmission distance. Various wireless communication methods used in the control systems have their own advantages and disadvantages, but the common shortcoming is that they are only suitable for behavior control in small-scale environments. Communication methods relying on base stations for data transmission have less limitation on transmission distance and are suitable for outdoor large-scale environmental control tasks, but the problems of high power consumption and transmission delay still need to be addressed.

In 2017, Su’s team [42] developed a device for controlling pigeon behavior in outdoor conditions. They achieved stimulation control of typical flight behaviors in pigeons and simultaneously recorded their flight trajectories based on GPS recording. In 2018, Dai’s team [6] designed a miniature wireless navigational stimulator that can be carried by pigeons. They realized long-distance outdoor flight control of pigeon robots over a specific time and location using a pre-programmed hierarchical stimulation algorithm. In 2019, Wan and Shang’s team [64] designed a wearable system for remote real-time behavior control of animal robots. They achieved flight control of pigeons and effectively solved the problem of short communication distances in current control systems based on the general packet radio service (GPRS) network communication. In 2020, Dai’s team [7] continued to use the mentioned pre-programmed hierarchical stimulation algorithm to achieve long-distance outdoor flight control of pigeon flocks for the first time, which has important implications for the study of pigeon flight control from the laboratory to the outdoors. In 2021, Wang and Liu’s team from HHU [65] developed a remote neural stimulator with a signal generation module, communication module, and positioning module distributed installation. The distributed design effectively alleviated the weight pressure on the pigeons and ensured the effectiveness of outdoor control during pigeons’ long-distance flight.

In practical application scenarios where the control environment is becoming increasingly complex, the design of reasonable and effective long-distance communication solutions and the development of miniature lightweight control systems are still bottlenecks that need to be overcome for outdoor pigeon flight behavior control research. Therefore, considering the wireless communication solutions mentioned above, it is still advisable to use civilian communication networks including GPRS, 3G, 4G, and 5G [54,64] to achieve long-distance signal transmission for pigeon flight control tasks in large-scale and complex outdoor environments. To further extend working time and reduce the burden on pigeons, distributed wearable miniature control systems with low power consumption and small packaging characteristics still need to be further studied and developed.

### 3.4. Flock-Oriented Control

Pigeons often use coordinated flight in groups to perceive external information and quickly propagate individual responses within the cluster. This natural and efficient control strategy ensures that the group exhibits high orderliness and robustness, which guarantees high flight efficiency [66]. Therefore, biomimetic research inspired from this strategy has important application value in multi-UAV coordination systems to complete specific tasks and multi-agent control systems. However, current research mainly focuses on individual pigeons (Table 2, column ‘Subjects’). The neglect of pigeon flock flight characteristics means that current pigeon robots cannot make good use of their extraordinary group coordination ability.

In 2020, Dai’s team [7] expanded the scope of altering pigeon motor behavior using brain stimulation at the group level. They used a pre-programmed hierarchical stimulation algorithm to implement artificial control of high-level leader pigeons in the flock. This study achieved long-distance control of the overall flight status of a pigeon flock for the first time, and is a beneficial attempt in pigeon robot research to move towards flock-oriented control. In 2021, Hou and Wang’s team from ZZU [67] analyzed the flying characteristics of pigeon flocks and achieved flight behavior control of a flock including leader and subordinate pigeons using wireless remote control and intragroup self-organizing network communication.

Currently, research on pigeon flock flight behavior control is still in its early stage. Individual pigeon robots are limited by their detection, positioning, and perception capabilities, which hinders their ability to be applied in broader scenarios. Therefore, there is still room for breakthroughs in flock-oriented pigeon robot research. In flock behavior, individual pigeons often adhere to simple rules without exhibiting overly complex behaviors. Additionally, individual behaviors can be influenced by the behavior and state of the flock, and they can also influence the flock behavior to some extent [68]. This means that complex flock behavior is not simply the sum of individual behaviors. The distributed, self-organizing, and highly adaptive behavioral patterns displayed in the flock pose challenges to flock-oriented pigeon robot research. In future studies, there will be an increasing need for research on the intrinsic mechanisms of flock behavior through experiments and model analysis. The study of efficient information transmission mechanisms among individuals in the flock, and the simulation of flock behavior applied to the control of pigeon flock flight behavior, are also promising future directions.

### 3.5. Multi-Functionality of Control Systems

In early research on the control of pigeon motion, the control system typically only included essential microstimulators, power modules, and communication modules. However, pigeon robots have been rapidly developed and their applications have become more diverse in recent years. The diversified information acquisition components that record multiple types of data have been added to bird-loaded systems. These data types include temperature and acceleration data [43], GPS data [64], neural signal data [24], pulse signal and imaging data [69], speed data [70], electromyography (EMG) data [23,71], and fused posture data [72]. This has led to the development of control systems that integrate multiple types of information acquisition, moving toward multifunctional capabilities (Table 2, column “Functions”). Nevertheless, research that combines monitoring of behavioral and neural data with stimulation control equipment to achieve closed-loop control of pigeon robots is still limited.

Due to the importance of deeply understanding the encoding patterns of neural electrical signals in specific brain regions, it is crucial to set appropriate stimulation parameters and achieve precise and specific control. Therefore, there is a pressing need to develop bird-loaded devices that combine stimulation control with neural signal recording capabilities, especially for real-time closed-loop control needs. Eichenbaum et al. [73] developed the first miniature wearable neural signal detection and recording system based on wireless data transmission technology in 1977. They successfully recorded spike potentials and local field potential signals within specific brain regions. Subsequent studies [74,75] designed and developed data recording systems suitable for birds, but they all had varying degrees of drawbacks such as large size, high power consumption, low accuracy, or short transmission distance. In 2016, Rattenborg et al. [44] used a customized neural signal acquisition chip to record the multi-channel neural signals of albatrosses during their 10-day flight over the ocean along with their three-dimensional acceleration data. In 2018, Wan and Shang’s team [24] designed a neural signal recording and stimulation control system for pigeons. This system can collect pigeons’ neuroelectrophysiological signals and apply electrical stimulation to the target nucleus based on real-time signal processing and analysis, thereby achieving closed-loop control of pigeon motion based on electrophysiological recording.

### 3.6. Future Prospects and Challenges

The diversified information about the external environment, spatial position, animals’ physiological state, and autonomous motion intentions obtained by the above components provide possibilities for dual-loop or even multi-loop control. However, it is challenging to establish unified control strategies and set priorities for stimulation commands due to the potentially complex nature of the information involved and further exploration of the implementation is still required. Additionally, although the availability of devices for obtaining diverse information expands the functionality of control systems, it also presents challenges to the weight-bearing capacity of pigeons [76] and the concealment of implanted devices [25,77]. Therefore, it is important to analyze the weight-bearing and force characteristics of birds to design wearable systems suitable for distributed load-bearing in pigeons in future research. Developing highly miniaturized, integrated, and concealed bird-loaded systems will help alleviate the limitations imposed by weight-bearing capacity on the upgrade and advancement of multifunctional control systems.

**Table 2:** Overview of main research advances of altering pigeon motor behavior using brain stimulation since 2007.

Institution	Year	Contribution	Targets	Parameters	Environments	Subjects	Functions
SDUST	2007	The world’s first “robot bird” [5]	DIVA, PoA	current amplitude, pulse frequency, width and pulse train number	indoor	individual	stimulation
	2012	A common brain mechanism of motor behaviors control and control method [8]	DIVA, PoA	current amplitude, pulse frequency and width	indoor	individual	stimulation
	2015	A new multi-mode telestimulation system for brain-microstimulation for robo-pigeon navigation [38]	DIVA, PAG	current amplitude, pulse frequency, duration and pulse train number	indoor	individual	stimulation
	2016	Robo-pigeon based on multiple brain region synchronization using implanted microelectrodes [21]	DIVA, archistriatum	——	indoor	individual	stimulation
	2017	A GPS-based stimulator enabling quick and clear flight control evaluation of the robo-pigeon in open space [42]	DIVA, PAG	voltage amplitude, pulse frequency, duration and pulse train number	outdoor	individual	stimulation; GPS and velocity recording
	2018	Controlling robo-pigeon walk forward and backward along a prescribed straight line based on ICo microstimulating [47]	ICo, DIVA	current amplitude, pulse frequency and duration	indoor	individual	stimulation
	2022	Online monitoring system of photovoltaic cells for pigeon-robot stimulators [20]	DIVA	voltage amplitude, frequency, duration and duty cycle	outdoor	individual	stimulation and photovoltaic cell monitoring
		A novel rechargeable sEMG acquisition system for animal robot [71]	PAG	voltage amplitude and frequency	indoor	individual	stimulation and EMG recording
	2023	Magnetic stimulation coil for robot pigeons [23]	——	magnetic field amplitude	indoor	individual	stimulation and EMG recording
NUAA	2012	Inducing wing flapping, shortness of breath by electrically stimulating midbrain of pigeon [39]	Tos, ICo, SGP	current amplitude, pulse frequency, width and duration	indoor	individual	stimulation
	2014	Remote control equipment for pigeon robots flying outdoors [69]	Tos, ICo	current and voltage amplitude, pulse frequency and width	indoor	individual	stimulation; GPS, pulse and image recording
	2015	Pigeon control targeted in midbrain motor regions [10]	ICo, FRM, LoC	current amplitude, pulse frequency and duration	indoor	individual	stimulation
	2018	Outdoor long-distance flight control of robo-pigeons [6]	ICo, FRM	voltage amplitude, pulse number, frequency and duration	outdoor	individual	stimulation and GPS recording
	2020	Flying pigeon flock control [7]	FRM	current amplitude	outdoor	flock	stimulation and GPS recording
	2022	Steering behavior control of pigeon robot based on preset straight path [48]	——	voltage amplitude, pulse frequency and width	outdoor	individual	stimulation and GPS recording
		A new control system for pigeon robots integrating the function of trajectory monitoring and stimulation [70]	FRM	voltage amplitude, pulse frequency and width	outdoor	flock	stimulation and trajectory monitoring
	2023	Grade-control outdoor turning flight of robo-pigeons with quantitative stimulus parameters [22]	FRM	current frequency, duration, and interval	outdoor	individual	stimulation and GPS recording
SNU	2017	A polymer-based fully implantable wireless stimulator for pigeons using depth electrodes [11]	FRM	current amplitude, pulse frequency and duration	indoor	individual	stimulation
	2020	Fully-implantable stimulation system for remote avian navigation [17]	FRM	current amplitude, pulse frequency and duration	indoor	individual	stimulation
	2021	Avian flight control based on midbrain stimulation [18]	RT, OM, TN, TSM, AV	current amplitude, pulse frequency and duration	indoor	individual	stimulation
		Pigeon-robot implanted the biofuel cell and brain stimulator [19]	——	——	indoor	individual	stimulation
ZZU	2018	Pigeon wearable neural signal detection and stimulation closed-loop control system [24]	FRM	current amplitude, pulse frequency, interval, duty cycle and pulse train number	outdoor	individual	stimulation and neural signal recording
	2019	Multi-parameter microstimulation for pigeon flight modulation [16]	FRM	current amplitude, pulse frequency and duty cycle	indoor	individual	stimulation
		A wearable behavior control system for robo-animals [64]	FRM	current amplitude, pulse frequency, duration and duty cycle	outdoor	individual	stimulation and GPS recording
	2021	A pigeon robot remote control system with the function of stimulation and parameter optimization [57]	DIVA, PoA	current amplitude, pulse frequency and duration	indoor	individual	stimulation
		A networked cluster flight control system for robot-pigeons [67]	——	pulse amplitude	outdoor	flock	stimulation and GPS recording
	2022	PoA is an important brain area that mediates pigeon turning behavior [37]	PoA	current amplitude, pulse frequency and interval	indoor	individual	stimulation
		Locomotion control of pigeon robots based on virtual fear using micro-stimulation [40]	SGP	current amplitude, pulse number, frequency, interval, duty cycle and pulse train number	outdoor	individual	stimulation and GPS recording
	2023	Electrical stimulation of the SGP induced evasive behavior in pigeons away from their original homing route [41]	SGP	current amplitude, pulse number, frequency, width and interval	outdoor	individual	stimulation and GPS recording
HHU	2021	A long-range neural stimulator for pigeon robots [65]	FRM	current amplitude, pulse number, frequency and duty cycle	outdoor	individual	stimulation and GPS recording
	2023	Wireless-controlled cubic neural stimulator for free-moving animals [49]	FRM	current amplitude, pulse frequency and phase-width ratio	indoor	individual	stimulation
		Embedded neural stimulator based on flexible printed circuit board [25]	FRM	current amplitude, pulse number and frequency	outdoor	individual	stimulation and GPS recording

Note. DIVA, dorsalis intermedius ventralis anterior. PoA, posterior pallial amygdala. ToS, torus semicircularis. ICo, nucleus intercollicularis. SGP, substantia griseaet fibrosa periventricularis. FRM, formatio reticularismedialis mesencephali. LoC, locus ceruleus. PAG, periaqueductal gray. RT, nucleus rotundus. OM, tractus occipito-mesencephalicus. TN, nucleus taeniae. TSM, tractus septo-mesencephalicus. AV, archistriatum ventral. GPS, global positioning system. EMG, electromyography.

## 4. Summary

A popular subject in cyborg and bionic systems, altering pigeon motor behavior using brain stimulation is a core research topic in the field of pigeon robotics. It involves multiple interdisciplinary fields including animal behavior, neuroscience, electrical information technology, and artificial intelligence. Based on a survey and analysis of relevant literature in recent years, we first introduced the neural basis and system composition of altering pigeon motor behavior using brain stimulation in this paper. Furthermore, we classified and reviewed representative research results in this field in recent years, summarizing and discussing the progress of relevant research in terms of control targets, parameters, environments, subjects, and systems involved. Finally, we also provided an outlook on the future development trends in research on altering pigeon motor behavior using brain stimulation, trying to highlight the developing directions of the next stage in this field.

## Figures and Tables

**Table 1:** The control strategies of pigeons’ motion.

Control Strategy	Common Targets	Induced Behavior	Reference
Specific regions in the sensory system as the target along with behavioral training	HA	preening feathers with beak	[8]
	HD		
Specific regions in the motivation and emotion system or pallium as the target to induce motives or virtual emotions	DIVA	takeoff, left/right turn, avoidance	[5,8,37,38]
	PoA		
	PAG		
Specific regions in the midbrain motor regions as the target to directly control motion	ToS	wing-flapping, feather-lodging, left/right turn, forward motion, takeoff	[10,18,39,40,41]
	ICo		
	SGP		
	FRM		
	LoC		
	RT, OM, TN, TSM, AV		

Note. HA, hyperstriatum accessorium. HD, hyperstriatum dorsal. DIVA, dorsalis intermedius ventralis anterior. PoA, posterior pallial amygdala. PAG, periaqueductal gray. ToS, torus semicircularis. ICo, nucleus intercollicularis. SGP, substantia griseaet fibrosa periventricularis. FRM, formatio reticularismedialis mesencephali. LoC, locus ceruleus. RT, nucleus rotundus. OM, tractus occipito-mesencephalicus. TN, nucleus taeniae. TSM, tractus septo-mesencephalicus. AV, archistriatum ventral.

## Data Availability

Not applicable.
